# Nursing Interventions for Pain and Sleep Management in Cancer Patients Receiving Immune Checkpoint Inhibitors: A Scoping Review

**DOI:** 10.1002/nop2.71022

**Published:** 2026-11-10

**Authors:** Omar Alqaisi, Mohammed Dibas, Safia Samir Darwish, Amaechi Dennis, Patricia Tai

**Affiliations:** ^1^ Nursing Department Al‐Zaytoonah University Amman Jordan; ^2^ Department of Medicine, Faculty of Medicine and Health Sciences An‐Najah National University Nablus Palestine; ^3^ Nursing Program Batterjee Medical College Jeddah Saudi Arabia; ^4^ Department of Biochemistry/Medical Biochemistry Veritas College of Medicine, Veritas University Bwari Nigeria; ^5^ Department of Oncology University of Saskatchewan Saskatoon Saskatchewan Canada

**Keywords:** cancer, immune checkpoint inhibitors, nursing interventions, pain management, scoping review, sleep assessment, sleep disturbance, symptom management

## Abstract

**Aim:**

To map nursing interventions for pain and sleep disturbance among adults receiving immune checkpoint inhibitors and identify assessment and research gaps.

**Design:**

A scoping review.

**Review Methods:**

Eligible sources were charted using a standardized form and descriptively synthesized by intervention type, outcome, study design and evidence stream.

**Data Sources:**

PubMed, Scopus, ScienceDirect and the Cumulative Index to Nursing and Allied Health Literature were searched on 30 November 2025 for English‐language sources published from 1 January 2019 to 30 November 2025.

**Results:**

We included 10 sources: one randomized clinical trial, four systematic reviews, three of which included meta‐analysis, three observational studies, one mixed‐methods study and one structured clinical perspective/review. Four sources provided direct evidence from populations receiving immune checkpoint inhibitors, whereas six provided potentially transferable evidence from broader oncology populations. Electronic patient‐reported outcome monitoring produced promising findings in one immune checkpoint inhibitor trial. Broader oncology evidence identified potentially transferable pain‐management approaches. Two primary studies explicitly reported sleep‐related findings, but neither reported using a dedicated, validated sleep‐specific instrument. Sleep‐related content within broader symptom or quality‐of‐life assessments was distinguished from dedicated sleep assessment. No included ICI‐specific nursing intervention primarily targeted insomnia.

**Conclusion:**

Direct evidence for nurse‐led pain and sleep interventions during immune checkpoint inhibitor treatment remains limited. Findings from broader oncology populations should not be interpreted as evidence of effectiveness in this population.

**Implications for the Profession and/or Patient Care:**

Oncology nurses may consider assessing pain and sleep separately. Future research should incorporate validated sleep measures and explore interventions tailored to patients receiving immune checkpoint inhibitors.

**Impact:**

The study addressed the limited nursing evidence for managing pain and sleep disturbance during immune checkpoint inhibitor treatment. The main findings were the scarcity of direct intervention evidence and the absence of validated sleep‐specific assessment. The findings will inform oncology nurses, researchers and services caring for adults receiving immune checkpoint inhibitors.

**Reporting Method:**

The review followed the Preferred Reporting Items for Systematic Reviews and Meta‐Analyses Extension for Scoping Reviews.

**Patient or Public Contribution:**

No patients or members of the public were involved in this review, which was based on published evidence.

**Abbreviations**
AE: adverse event
CBT‐I: cognitive behavioural therapy for insomnia
CI: confidence interval
CTCAE: common terminology criteria for adverse events
ECOG: eastern cooperative oncology group
ED: emergency department
eHealth: electronic health
EORTC QLQ‐C30: European Organization for Research and Treatment of Cancer Quality of Life Questionnaire‐Core 30
ePRO: electronic patient‐reported outcome
HR: hazard ratio
HRQoL: health‐related quality of life
ICI: immune checkpoint inhibitor
IL: interleukin
irAE: immune‐related adverse event
OR: odds ratio
PD‐1: programmed cell death protein 1
PD‐L1: programmed death‐ligand 1
PRISMA‐ScR: Preferred Reporting Items for Systematic Reviews and Meta‐Analyses Extension for Scoping Reviews
PRO: patient‐reported outcome
PRO‐CTCAE: Patient‐Reported Outcomes Version of the Common Terminology Criteria for Adverse Events
PSQI: Pittsburgh Sleep Quality Index
QoL: quality of life
RCT: randomized controlled trial
SMD: standardized mean difference
TNF‐α: tumour necrosis factor alpha

## 1 Introduction

Cancer pain affects approximately 60% of patients with advanced malignancy, with nearly 20% experiencing moderate to severe pain that significantly impacts their quality of life and treatment adherence (Morikawa et al. 2023). Sleep disturbances present an equally significant burden, particularly in patients receiving immune checkpoint inhibitors (ICIs), a revolutionary cancer immunotherapy approved since 2011 that reinvigorates the host immune system to combat tumour cells, including melanoma (Dine et al. 2017). In a two‐center cross‐sectional study of 187 patients with advanced or metastatic cancer receiving immune checkpoint inhibitors, 51.9% had clinically relevant sleep disturbance, defined as a Pittsburgh Sleep Quality Index score > 5 and the mean score was 7.54 ± 5.39. Prevalence estimates reported for broader cancer populations range from 25% to 59% (Aktepe et al. 2025).

The clinical significance of these symptoms extends beyond patient comfort. Sleep disturbance is clinically relevant because it may occur alongside poorer performance status and greater disease and treatment burden. In a two‐center cross‐sectional study, an Eastern Cooperative Oncology Group performance status of 1 or higher, receipt of second‐line or later therapy and the presence of two or more metastatic sites were independently associated with sleep disturbance. These cross‐sectional associations do not establish temporality or causality (Aktepe et al. 2025; Balachandran et al. 2023). Specifically, the adjusted odds of sleep disturbance were higher among patients with an Eastern Cooperative Oncology Group performance status of 1 or higher (odds ratio 2.33, *p* = 0.015), those receiving second‐line or later therapy (odds ratio 2.43, *p* = 0.021), and those with two or more metastatic sites (odds ratio 2.10, *p* = 0.021) (Aktepe et al. 2025), similarly, inadequate pain management increases symptom burden and may interfere with patients' quality of life and engagement with care. Immune checkpoint inhibitors and other targeted anticancer therapies can cause diverse adverse effects that compromise treatment adherence and quality of life, highlighting the importance of nursing‐led patient education, toxicity monitoring and supportive care in addressing treatment‐related symptom burden (Alqaisi, Al‐Ghabeesh, et al. 2026). The need for timely recognition and management of treatment‐related toxicities is also evident in other immunotherapy settings, including CAR T‐cell therapy (Alqaisi, Dibas, et al. 2026).

Nursing interventions for cancer pain have been extensively studied (Liu et al. 2021), with a comprehensive scoping review identifying 22 types of nursing support from 72 studies (Morikawa et al. 2023). Education programs constitute the most researched intervention (31 studies), followed by physical interventions (massage, reflexology, exercises), cognitive behaviour approaches (Morikawa et al. 2023) and comfort nursing strategies that integrate physiologic, psychologic and social dimensions (Miao et al. 2022). However, benefit persistence varies by outcome and intervention type, and evidence from broader oncology populations does not show sustained effectiveness during ICI treatment (Park and Lee 2022). Long‐term sustainability remains controversial and unproven (Park and Lee 2022). More importantly, no included study evaluated an integrated nursing intervention designed specifically to address both pain and sleep disturbance among patients receiving ICIs. Pain and sleep disturbance may coexist and may involve overlapping inflammatory and symptom‐related pathways (Aktepe et al. 2025; Balachandran et al. 2023); however, this relationship requires further investigation in ICI‐treated populations (Bu et al. 2025).

Current patient‐reported outcome (PRO) instruments inadequately capture ICI‐specific symptoms, with 29% of adverse events remaining uncovered/unaddressed (Colomer‐Lahiguera et al. 2020). Instruments used to report PRO symptom‐related toxicities covered 45% of the most frequently reported AEs, whereas 23% of AEs were partially covered and 29% were not covered at all. Of non‐covered AEs, 59% referred to the dermatologic system. Partially covered AEs related to endocrine and specific types of pain (Colomer‐Lahiguera et al. 2020). Although electronic symptom‐monitoring approaches may facilitate early symptom detection and clinical follow‐up, their application to integrated pain and sleep management during ICI treatment remains insufficiently studied (Zhang et al. 2022; Kwok et al. 2022; Tolstrup et al. 2020). Specialized oncology nurses contribute to ICI care through patient education, symptom assessment, monitoring, triage and supportive follow‐up; however, evidence regarding implementation in community and non‐specialized healthcare settings remains limited (Mirzadeh et al. 2025).

The nursing relevance of this review lies in the central role of oncology nurses in the longitudinal assessment, triage, education and follow‐up of patients receiving ICIs (Mirzadeh et al. 2025). Nurses are often among the first clinicians to identify pain, sleep disruption, treatment‐related symptoms and barriers to self‐management of symptoms (Mirzadeh et al. 2025; Arbour et al. 2024; Brink 2024). However, nursing practice currently lacks ICI‐specific evidence to guide the routine assessment and integrated management of pain and sleep disturbance. Mapping the available direct and transferable evidence is therefore necessary to clarify what can be applied cautiously in current practice and to identify priorities for ICI‐specific nursing research.

Using a Population–Concept–Context (PCC) framework, this scoping review aimed to map nursing interventions for pain and sleep management among adult cancer patients receiving immune checkpoint inhibitors (Population), describe intervention characteristics and reported outcomes (Concept), across oncology clinical settings (Context) and to examine the extent to which sleep quality has been assessed or targeted by such interventions. The primary review question was: *‘What nursing interventions have been reported for managing pain and sleep disturbance in adult cancer patients receiving immune checkpoint inhibitors, and what evidence gaps remain*?’ Secondary objectives were to characterize technology‐based approaches, examine integrated symptom‐management models, and highlight gaps requiring future investigation.

## 2 Materials and Methods

### 2.1 Study Design

This scoping review was conducted and reported in accordance with the PRISMA‐ScR (Preferred Reporting Items for Systematic Reviews and Meta‐Analyses extension for Scoping Reviews) guidelines (Tricco et al. 2018). This review protocol was not prospectively registered, consistent with PRISMA‐ScR guidance that registration is not required for scoping reviews; this is noted as a limitation. The PRISMA checklist is presented in Table S1.

### 2.2 Search Strategy

A single combined electronic search was conducted in PubMed/MEDLINE, CINAHL, Scopus and ScienceDirect. The search combined terms related to nursing care, cancer, treatment context and pain or sleep outcomes. The treatment‐context block included ‘immunotherapy’, ‘immune checkpoint inhibitor’ and the broader term ‘cancer treatment’ to identify both direct evidence from immune checkpoint inhibitor treated populations and potentially transferable evidence from broader oncology populations.

The search was limited to English‐language publications from 1 January 2019 to 30 November 2025. This time frame was selected a priori to capture contemporary nursing evidence relevant to current immune checkpoint inhibitor treatment pathways. All cancer types were eligible to enable mapping across oncology settings. The complete database‐specific search strategies, applied limits and records retrieved from each database are presented in Table S2. The review aimed to map the breadth, characteristics and gaps in the available evidence; therefore, a prospectively registered protocol and formal critical appraisal were not undertaken. The final search was conducted on 30 November 2025.

### 2.3 Inclusion and Exclusion Criteria

A broader eligibility approach was adopted after an initial exploratory search restricted to ICI‐specific populations retrieved an insufficient volume of literature to characterize nursing intervention practice comprehensively; the transferability criteria were therefore defined prior to formal full‐text screening but after the exploratory search, and this sequence is reported here for transparency. Accordingly, this review was redefined to encompass two distinct evidence streams, which are analysed and reported separately throughout the Results and Discussion: (1) direct evidence from studies conducted in ICI‐treated populations, and (2) transferable evidence from studies conducted in broader oncology populations, judged transferable to ICI‐related nursing care based on mechanism‐of‐action/symptom‐management principles, nursing delivery model and symptom overlap.

Eligible sources included quantitative, qualitative and mixed‐methods primary studies; systematic reviews and meta‐analyses; and structured clinical perspective or review articles that presented an explicit nursing framework for patient education, assessment, monitoring, symptom management or support. Sources were required to address adults with cancer receiving immune checkpoint inhibitors or broader oncology populations whose findings met the predefined transferability criteria. Eligible outcomes included pain, sleep disturbance, symptom monitoring, patient‐reported outcomes, quality of life or closely related nursing outcomes. Only English‐language sources published between January 2019 and November 2025 were eligible.

Sources addressing PROs or HRQoL were included for assessment mapping when they provided information relevant to symptom assessment and monitoring in ICI care, even if they did not evaluate an explicitly nurse‐led intervention. Such sources informed the description of measurement approaches and assessment gaps rather than conclusions about nursing‐intervention effectiveness.

To improve transparency, transferable studies were considered eligible only when all of the following conditions were met: (1) the intervention was delivered or coordinated by nurses, or the source described a structured symptom‐assessment process undertaken by nurses; (2) the intervention or assessment process addressed pain, sleep disturbance, symptom monitoring or a closely related patient‐reported symptom outcome; (3) the intervention was feasible within oncology care for patients receiving systemic anticancer treatment; and (4) its components did not depend on a chemotherapy‐specific clinical pathway that would preclude cautious application in ICI care. Transferable evidence was not interpreted as evidence of effectiveness in ICI‐treated populations and was synthesized separately from direct ICI‐specific evidence.

Studies were excluded in the following cases: they did not meet the nursing‐intervention, nursing‐care‐framework or assessment‐mapping requirements described above; they did not address pain, sleep, symptom monitoring, PROs, HRQoL or another eligible nursing‐relevant outcome; they focused exclusively on pharmacological management without eligible assessment‐mapping content; they were conference abstracts, protocols, editorials or unstructured narrative discussions without eligible nursing or assessment content; or their populations and findings were not applicable to the review context. The structured clinical perspective/review by Mirzadeh et al. was retained because it provided an explicit framework for nursing education, assessment, monitoring and support during ICI treatment (Mirzadeh et al. 2025). It was classified separately from primary research and systematic reviews and was not interpreted as evidence of intervention effectiveness.

No minimum follow‐up duration was specified in the inclusion criteria, as this scoping review aimed to map the breadth of available evidence, regardless of study duration. However, follow‐up periods were systematically extracted and documented to assess the existing evidence base for the long‐term sustainability of benefits from nursing interventions.

### 2.4 Information Sources and Search Scope

No supplementary citation searching (backward/forward reference checking) or grey‐literature searching was undertaken; database searching was restricted to PubMed/MEDLINE, Scopus, ScienceDirect and CINAHL, which is acknowledged as a limitation potentially affecting the comprehensiveness of the evidence base.

### 2.5 Study Selection

Two reviewers independently screened titles and abstracts and subsequently assessed potentially eligible full texts. Disagreements were resolved through discussion and re‐examination of the source, with a third reviewer available for arbitration when consensus could not be reached.

### 2.6 Data Extraction

Two reviewers independently charted data using a standardized form that captured source characteristics, population, study design, evidence stream, intervention components, sample size, outcome measures and reported findings. Discrepancies were resolved through discussion and, when necessary, third‐reviewer arbitration.

Data extraction was conducted using a standardized form to capture study characteristics (author, year, country, population, disease stage), nursing intervention types and components, study design and setting, sample size, clinical and patient‐reported outcomes.

### 2.7 Critical Appraisal

A formal risk‐of‐bias assessment was not undertaken because the purpose of this scoping review was to map the breadth, characteristics and gaps of the available evidence rather than to evaluate the methodological quality or pooled effectiveness of individual studies. This approach is consistent with PRISMA‐ScR guidance, which does not require critical appraisal unless it is relevant to the review objectives (Tricco et al. 2018). Data were synthesized descriptively to categorize intervention types, study characteristics, reported outcomes and evidence gaps. The data‐charting form was not formally pilot‐tested, which may have limited consistency during initial data charting; however, all extracted data were independently checked by two reviewers, and discrepancies were resolved by consensus.

## 3 Results

### 3.1 Study Selection

The searches identified 485 records across PubMed/MEDLINE, CINAHL, Scopus and ScienceDirect, including records potentially relevant to both direct immune checkpoint inhibitor evidence and transferable oncology evidence. After removal of 127 duplicates, 358 records underwent title and abstract screening, of which 307 were excluded. Full texts were sought for 51 reports, but two could not be retrieved. The remaining 49 reports underwent eligibility assessment; 39 were excluded, and 10 sources were included in the review (Figure 1).

**FIGURE 1:**
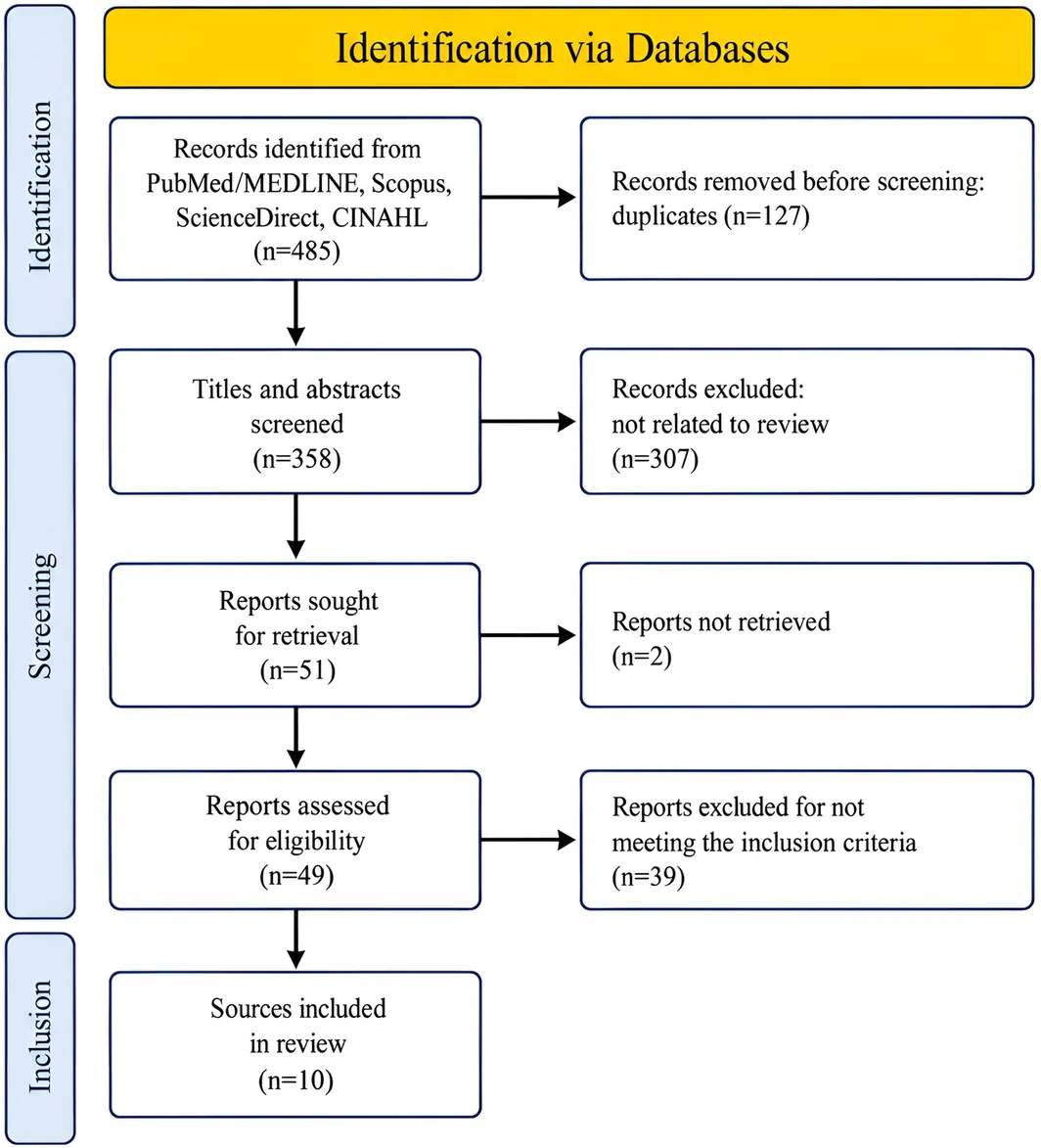
PRISMA 2020 flow diagram of the study selection process.

Reasons for exclusion following full‐text eligibility assessment are presented in Table S3. The two reports that could not be retrieved are reported separately and are not included among the 39 reports excluded after eligibility assessment.

### 3.2 Characteristics of Selected Studies

Of the 10 included studies (Park and Lee 2022; Bu et al. 2025; Zhang et al. 2022; Kwok et al. 2022; Tolstrup et al. 2020; Mirzadeh et al. 2025; Hall et al. 2019; Li et al. 2023; Yan et al. 2025; Liu and Guo 2025), 4 (40%) were ICI‐specific (Zhang et al. 2022; Tolstrup et al. 2020; Mirzadeh et al. 2025; Hall et al. 2019), while 6 (60%) examined general cancer populations with interventions deemed transferable to ICI contexts (Park and Lee 2022; Bu et al. 2025; Kwok et al. 2022; Li et al. 2023; Yan et al. 2025; Liu and Guo 2025). This classification (direct ICI evidence vs. transferable oncology evidence) is indicated for each study and is used to structure the presentation of findings.

The 10 included sources comprised one randomized clinical trial, four systematic reviews, three of which included meta‐analysis (Park and Lee 2022; Kwok et al. 2022; Hall et al. 2019; Yan et al. 2025), three observational studies, one mixed‐methods study and one structured clinical perspective/review. Four sources provided direct evidence from immune checkpoint inhibitor populations, whereas six provided potentially transferable evidence from broader oncology populations (Park and Lee 2022; Bu et al. 2025; Kwok et al. 2022; Li et al. 2023; Yan et al. 2025; Liu and Guo 2025). Sources represented several national contexts and multinational evidence syntheses. Table 1 summarizes the characteristics of the included sources, encompassing clinical trials and other types of evidence.

**TABLE 1:** Characteristics of included sources.

Authors/years	Country	Study design	Settings	Cancer type	Evidence stream	Evidence level	Sample size	Age (years)	ICIs used
Park and Lee (2022)	South Korea	Systematic review	Multiple databases	Various cancer type	Transferable broader oncology evidence	Secondary study: systematic review and meta‐analysis	22 RCTs	44.1–67.3	Music, physical, psycho‐educational
Bu et al. (2025)	China	Prospective longitudinal observational study	Tertiary hospital	Hepatocellular carcinoma	Transferable broader oncology evidence	Primary observational evidence	*N* = 130	46–69 (66.2%)	Immune + targeted + interventions
Zhang et al. (2022)	China	Open‐label, multicenter randomized clinical trial	28 Tertiary hospitals	Gastric, oesophageal, lung	Direct ICI‐specific evidence	Primary randomized clinical trial	*N* = 278 analysed; intervention *n* = 141, control *n* = 137	58.8 ± 12.7	Multiple ICIs
Kwok et al. (2022)	Multiple countries	Systematic review	Multiple settings	Various cancer types	Transferable broader oncology evidence	Secondary study: systematic review and meta‐analysis	2315 (10 studies)	Variable means	Various (review)
Tolstrup et al. (2020)	Denmark	Mixed method study	University hospital	Metastatic *melanoma*	Direct ICI‐specific evidence	Primary mixed‐methods study	*N* = 70 (57 surveyed)	Median 65	Immunotherapy
Mirzadeh et al. (2025)	Canada	Structured clinical perspective/review	Clinical perspective evidence	All ICI candidates	Direct ICI‐specific evidence	Perspective/Review article	Review	Not specific	PD‐1/PD‐L/CTLA‐4
Hall et al. (2019)	USA/Multi country	Systematic review	Multicenter trials	*Melanoma*, lung, GU, H&N	Direct ICI‐specific evidence	Secondary study: systematic review	15 RCTs	44.1–67.3	Nivolumab, pembrolizumab
Li et al. (2023)	China	Retrospective comparative study	2 tertiary hospitals	Colorectal cancer	Transferable broader oncology evidence	Primary observational evidence	*N* = 100 (50 int.)	Not specified	Adjuvant/Chemotherapy
Yan et al. (2025)	China	Systematic review	Multiple databases	Various cancer types	Transferable broader oncology evidence	Secondary study: systematic review and meta‐analysis	1070 (17 RCTs)	Not specific	Various (review)
Liu and Guo (2025)	China	Retrospective cohort	Hubei cancer hospital	Lung cancer (Stage II–IV)	Transferable broader oncology evidence	Primary retrospective cohort	Initial cohort: *N* = 291 (137 intervention, 154 control); *N* = 252 after propensity‐score matching. Several outcome tables retained the original group sizes.	65.1 ± 7.9 vs. 65.5 ± 8.4	Chemo, targeted, immune

Abbreviations: CRC, colorectal cancer; CTLA‐4, cytotoxic T‐lymphocyte‐associated protein 4; GU, genitourinary; HCC, hepatocellular carcinoma; H&N, head and neck; ICI, immune checkpoint inhibitor; int., interventions group; *N*, sample size; PD‐1, programmed cell death protein 1; PD‐L1, programmed death‐ligand 1; RCT, randomized controlled trial.

Sample sizes are reported separately by study type to avoid double counting. Primary‐source sample sizes are reported individually because enrollment and analysis populations were not consistently defined across reports. Zhang et al. (2022) analysed 278 participants; Bu et al. (2025) reported 130 participants; Li et al. (2023) reported 100 participants; and Tolstrup et al. (2020) enrolled 70 participants, of whom 57 completed the survey. Liu and Guo (2025) reported an initial cohort of 291 participants and a propensity‐score‐matched sample of 252, while several outcome tables retained the original group sizes. No combined participant total was calculated across these heterogeneous reports. The reviews by Hall et al. (2019), Yan et al. (2025), Kwok et al. (2022) and Park and Lee (2022) included 15, 17, 10 and 22 studies, respectively, for a total of 64 review‐level study inclusions.

Because potential overlap among their primary studies was not assessed, this aggregate should not be interpreted as 64 unique studies or combined with participant totals from the primary sources. Because primary studies may overlap across reviews and may not be independent of the primary studies included directly in this scoping review, no combined total sample size across all 10 included sources is presented.

### 3.3 Nursing Intervention Types and Components

Descriptive categorization identified five groups of nursing interventions: educational, physical, cognitive‐behavioural, integrated symptom management and technology‐based. Intervention characteristics are detailed in Table 2.

**TABLE 2:** Intervention details and populations.

Study	Cancer stage	Intervention type	Key components	Duration	Delivery methods
Park and Lee (2022)	Various (systematic review)	Nurse‐led nonpharmacological interventions	Music interventions, physical exercises, psycho‐educational programs	Various timeframes	Various (nursing delivered)
Bu et al. (2025)	Advanced stage	Symptom self‐report questionnaires	Symptoms assessment at Weeks 1,2 and 3 using standardized scales	3 weeks post intervention	Paper‐based questionnaires (in‐person)
Zhang et al. (2022)	Mixed cancer types	ePRO follow up model with alerts	Questionnaire + image recognition for irAE grading; automated advice for grades 1–2; alert for grades 3–4	6 months or until treatment ends	Mobile app/web‐based + image recognition
Kwok et al. (2022)	Various	Nurse‐led telehealth	Telephone calls, video consultations, web‐based systems, SMS, mobile apps (reactive/scheduled)	≥ 4 weeks minimum	Telephone, video, web SMS, mobile applications
Tolstrup et al. (2020)	Metastatic	eHealth PRO weekly	Weekly PRO‐CTCAE symptoms reporting via tablet from home	Weekly during treatment	eHealth platform (home‐based)
Mirzadeh et al. (2025)	Various stages	Nursing education, assessment and monitoring	Patient education (multiple formats), symptoms assessment, nurse‐led clinics, support services	Ongoing throughout treatment	In‐person telephone, video, written materials
Hall et al. (2019)	Unresectable/Metastatic (*n* = 13), adjuvant (*n* = 2)	PRO measurement	Systematic review of 15 ICI trials with PRO data	Various	Various clinical trial questionnaires
Li et al. (2023)	Various	Pain education nursing with mind mapping	Nurses used mind map to guide pain education and perioperative care planning	Perioperative and postoperative	Repeated in‐person education sessions
Yan et al. (2025)	Various (systematic review)	Non‐pharmacological pain management	Reflexology, aromatherapy, acupressure, massage therapy, acupuncture.	Varied (1990–2023)	In‐person therapy sessions
Liu and Guo (2025)	Stage II–IV (72.3% stages III–IV)	Personalized nursing care	20–30 min baseline consultation + telephone/video follow‐ups on Days 4 and 10 of each cycle	8 weeks post‐treatment initiation	Telephone and video consultations

Abbreviations: CRC, colorectal cancer; eHealth, electronic health; ePRO, electronic patients reported outcome; HCC, hepatocellular carcinoma; ICI, immune checkpoint inhibitor; irAE, immune related adverse event; PRO, patients‐reported outcome; PRO‐CTCAE, patients reported outcomes version of the common terminology criteria for adverse events; SMS, short message service.

### 3.4 Educational Interventions

Educational components were described across six included sources (Park and Lee 2022; Zhang et al. 2022; Kwok et al. 2022; Mirzadeh et al. 2025; Li et al. 2023; Liu and Guo 2025). Individualized 20–30‐min consultations incorporated baseline quality of life screening, symptom anticipation counselling and personalized care planning (Liu and Guo 2025).

Mind mapping is a combination of images and logical thinking in the form of pictures and text. It was used as a structured teaching tool to present pain education and perioperative care precautions (Li et al. 2023). Nurses created and used it to structure pain‐education content. Mind maps visually organize:

- Pain mechanisms
- Expected postoperative pain trajectory
- Analgesic options
- Nonpharmacological pain strategies
- Self‐management steps

Park and Lee (2022) identified psychoeducational interventions as one category of nurse‐led nonpharmacological care in broader oncology populations. Their review distinguished combined estimates across outcome domains from analyses by intervention type and individual outcome. Quantitative findings are presented under Reported outcomes to avoid conflating these analyses.

#### 3.4.1 Physical Interventions

Yan et al. (2025) conducted a systematic review and meta‐analysis of 17 randomized controlled trials evaluating nonpharmacological nursing approaches to cancer pain management. Massage therapy was associated with reduced pain in broader oncology populations (standardized mean difference −1.00, 95% confidence interval −1.08 to −0.92; *p* < 0.001), with negligible heterogeneity (*I* ^2^ = 0%). Acupuncture yielded a larger pooled effect (standardized mean difference −2.09, 95% confidence interval −2.92 to −1.26; *p* < 0.001), but heterogeneity was high (*I* ^2^ = 88%). These estimates were derived from broader oncology populations and should not be interpreted as immune checkpoint inhibitor–specific effectiveness (Yan et al. 2025). Reflexology, aromatherapy and acupressure also significantly reduced cancer‐related pain (all *p* < 0.001) (Yan et al. 2025).

### 3.5 Cognitive‐Behavioural and Integrated Interventions

In the abstract of their retrospective lung cancer cohort report, Liu and Guo (2025) reported that personalized nursing was associated with a greater mean improvement in EORTC QLQ‐C30 scores than standard care (13.2 ± 7.6 vs. 5.1 ± 6.8 points; *p* < 0.001). The authors also reported higher treatment adherence (91.2% vs. 78.6%; *p* = 0.006) and less frequent unplanned healthcare use (12.4% vs. 23.4%; *p* = 0.014). The source report contains inconsistencies in outcome reporting and analysis‐population denominators across sections; these findings were therefore interpreted cautiously. In the longitudinal observational study by Bu et al. (2025), pain prevalence decreased from 73.8% to 48.5% over 3 weeks. Because Bu et al. (2025) lacked a controlled comparison, this review did not treat the temporal change as evidence of a nursing‐intervention effect.

### 3.6 Technology Based Approaches

Tolstrup et al. (2020) examined patients' and clinicians' experiences with electronic symptom reporting during immunotherapy and highlighted the importance of incorporating submitted reports into clinical consultations. Zhang et al. (2022) evaluated an ePRO follow‐up model combining weekly symptom reporting, automated advice and alerts to a clinical team that included oncology nurses. The trial's safety and healthcare‐use findings are presented under Health services utilization. Kwok et al. (2022) reviewed nurse‐led telehealth approaches delivered through telephone, video, web‐based and mobile platforms in broader oncology populations.

### 3.7 Reported Outcomes

Table 3 shows recent studies in the past 7 years. In a meta‐analysis of broader oncology populations, nurse‐led nonpharmacological interventions were associated with a moderate short‐term reduction in pain (Hedges' *g* = 0.39, 95% CI 0.25–0.52, *p* < 0.001) (Park and Lee 2022). Long‐term pain effects were substantially diminished (*g* = 0.10; 95% CI: −0.05–0.26; *p* = 0.187), except for psycho‐educational interventions which maintained small but significant effects (*g* = 0.25, *p* = 0.022) (Park and Lee 2022). All five physical intervention modalities significantly reduced cancer pain (*p* < 0.001), with acupuncture showing the largest effect (SMD −2.09) and massage showing the most consistent results (I2 = 0%) (Yan et al. 2025). Visual analog scale pain scores were significantly lower in pain‐education nursing groups (*p* < 0.05) (Li et al. 2023). Researchers are continually trying to address challenges in cancer pain management by improving nurses’ knowledge, and education on this topic should be included in the nursing training curriculum and continuous professional development after graduation (Saifan et al. 2019; Al Omari et al. 2021).

**TABLE 3:** Outcome measures, reported findings and classification of sleep assessment across included sources.

Study	Instruments used	Pain/Sleep outcome role	Sleep assessment classification	Primary outcomes	Future recommendations
Park and Lee (2022)	Various pain measure, HRQoL instruments	Pain reduction was the primary outcome	Review‐level reporting included insomnia among other physical symptoms assessed in component studies; this was not presented as a separate pooled insomnia outcome.	Pain reduction, knowledge of pain management, pain coping	Standardized intervention protocols; optimal dosing guidelines; mechanism studies; patient‐centered outcomes
Bu et al. (2025)	Symptom assessment scale (Likert 0–6)	Dynamic symptom burden; insomnia was one item within a broader symptom scale	Insomnia item within a broader symptom assessment; validation as a dedicated sleep measure was not reported.	Dynamic symptom changes over 3 weeks	Extended longitudinal studies; larger sample sizes; earlier intervention (pre‐treatment); trajectory modelling
Zhang et al. (2022)	PRO‐based QoL questionnaire, EORTC QLQ‐C30	Broader ePRO/irAE and QoL outcomes; pain/sleep not primary outcomes	General QoL assessment containing sleep‐related content; no separately reported sleep outcome identified in the charted trial findings.	Serious irAEs (Grades 3–4), ED visits, QoL, treatment discontinuation	Large‐scale RCTs in diverse populations; cost effectiveness studies; digital literacy interventions
Kwok et al. (2022)	EORTC QLQ‐C30, EQ‐5D, various symptom scales	Symptom severity and healthcare utilization; pain may be one component	Review‐level reporting of sleep‐problem improvement in a component study; not a separate pooled sleep‐effect estimate.	Health services use, QoL, symptom severity	More nurse‐led telehealth RCTs; consistent outcomes measurement; reactive vs. scheduled comparison; cost‐effectiveness
Tolstrup et al. (2020)	Patients feedback form (13 items), interviews, focus group	Broader symptom monitoring; pain/sleep not reported as primary outcomes	Not assessed as a sleep‐specific outcome	Patient/clinician satisfaction, symptom awareness, patient involvement	Standardize PRO measurement; improve clinician‐patients communication tracking; multicenter validation
Mirzadeh et al. (2025)	Risk assessment tools, educational frameworks	Narrative discussion of symptom assessment	Not assessed	Early detection of irAEs, the effectiveness of patient education	Multi‐setting evaluation of nursing roles; systematic protocols for early detection; international collaboration
Hall et al. (2019)	EORTC QLQ‐C30 (80%), EQ‐5D (67%), FKSI‐DRS, LCSS, EORTC QLQ‐H&N35	Broader HRQoL/PRO reporting; sleep may be included in some HRQoL measures	Broader HRQoL assessment, including instruments with sleep‐related content; not a dedicated sleep assessment.	HRQoL with ICIs vs. other therapies	Develop ICI‐specific PRO instruments; harmonize outcomes measurement; long‐term HRQoL tracking
Li et al. (2023)	VAS (pain), SAS/SDS (anxiety/depression), EORTC QLQ‐C30	Pain was a primary outcome	General QoL instrument containing sleep‐related content; sleep was not charted as a separate outcome.	Postoperative pain, QoL, emotional distress, comfort	Multicenter trials; long‐term follow‐up; blinded design; economic evaluation; adaptability to different settings
Yan et al. (2025)	BPI, NRS, VAS	Pain was the primary outcome	Not assessed	Cancer‐related pain reduction	Standardized intervention protocols; dose–response studies; combination therapy trials; 6–12‐month outcomes
Liu and Guo (2025)	EORTC QLQ‐C30; psychological distress/anxiety measures; reported sleep‐disturbance indicator; 0–10 sleep‐quality rating.	Overall QoL was the primary outcome; sleep disturbance and sleep‐quality ratings were also explicitly reported.	Explicit sleep‐disturbance reporting and a 0–10 sleep‐quality rating; no dedicated validated sleep instrument reported.	Overall QoL improvement at 8 weeks	Multicenter RCTs with extended follow‐up; diverse populations; cost–benefit analysis; mechanistic studies

*Note:* Sleep‐assessment classification distinguishes explicitly reported sleep findings from sleep‐related content embedded within broader symptom or HRQoL instruments. The presence of a sleep‐related item in a general instrument was not considered equivalent to dedicated sleep assessment. No dedicated, validated sleep‐specific measure was identified in the charted outcomes. This finding concerns the measures reported and charted for this review and should not be interpreted as proof that every primary study contained within the included evidence syntheses omitted such measures.

Abbreviations: BPI, brief pain inventory; ED, emergency department; EORTC QLQ‐C30, European organization for research and treatment of cancer quality of life questionnaire‐Core 30; EORTC QLQ‐H&N35, European organization for research and treatment of cancer quality of life questionnaire‐head and neck 35; EQ‐5D, Euro‐QoL 5‐dimension questionnaire; FKSI‐DRS, functional assessment of cancer therapy‐kidney symptom index‐disease related symptoms; HADS, hospital anxiety and depression scale; HRQoL, health‐related quality of life; ICI, immune checkpoint inhibitor; irAEs, Immune related adverse events; LCSS, lung cancer symptom scale; NRS, numeric rating scale; PRO, patients reported outcome; QoL, quality of life; RCT, randomized controlled trial; SAS, self‐rating anxiety scale; SDS, self‐rating depression scale; STAI, state–trait anxiety inventory; VAS, visual analog scale.

Sleep‐related evidence was distinguished according to whether a source reported an explicit sleep outcome, included sleep‐related content within a broader assessment instrument, or reported use of a dedicated sleep‐specific measure. Two included primary studies explicitly reported sleep‐related findings: Bu et al. (2025) and Liu and Guo (2025). Bu et al. (2025) reported insomnia within a broader symptom assessment, with prevalence decreasing from 53.1% in Week 1 to 44.6% in Week 3; they did not report validating a dedicated sleep‐specific measure. Liu and Guo (2025) reported sleep disturbance and a 0–10 sleep‐quality rating alongside broader quality‐of‐life and psychological outcomes. At the review level, Kwok et al. (2022) also reported improved sleep problems in a component study. Park and Lee (2022) also identified insomnia among the physical symptoms assessed in component studies, rather than as a separate pooled sleep outcome. These findings differed from the inclusion of sleep‐related items in general HRQoL instruments. The charted outcomes identified no dedicated, validated sleep‐specific instrument and no included ICI‐specific nursing intervention primarily targeted insomnia.

A cross‐sectional study found that sleep disturbance was associated with poorer performance status and greater treatment and disease burden among patients receiving immune checkpoint inhibitors; however, its design did not permit conclusions about survival prediction, temporality or causality. This relationship has been proposed to be mediated by elevated proinflammatory cytokines (IL‐1, IL‐6, TNF‐α), which are known to regulate sleep–wake homeostasis (Aktepe et al. 2025; Balachandran et al. 2023). These cytokines have also been linked to tumour progression in preclinical and observational models (Aktepe et al. 2025). Disruption of circadian rhythms in ICI‐treated patients has been hypothesized to influence immune function (Balachandran et al. 2023). Some evidence further suggests that sleep quality may be associated with immune checkpoint inhibitor efficacy (Zarogoulidis et al. 2023), although this remains an area requiring prospective investigation rather than an established causal mechanism.

While our study focused on immunotherapy patients, sleep disturbances and pain have been studied in the general cancer population (Sheikh‐Wu et al. 2020; Munir et al. 2025) with established interventions such as cognitive behavioural therapy for insomnia (CBT‐I) demonstrating large effect sizes (Nissen et al. 2024), and comprehensive symptom‐cluster research available across multiple cancer types (Sheikh‐Wu et al. 2020; Ye et al. 2025; Zhou et al. 2024; Liu et al. 2024). Ye, et al. from China reported 365 patients who completed questionnaires: symptom cluster Class 1 (‘low symptom burden’ class), Class 2 (‘fatigue‐insomnia’ class) and Class 3 (‘high symptom burden’ class), with a percentage of 54.5%, 38.6% and 6.8%, respectively (Ye et al. 2025). The quality‐of‐life score, introversion/extroversion, economic burden, Karnofsky Performance Status, albumin level and exercise self‐efficacy were significantly different among the 3 classes (*p* < 0.05). They concluded that exercise self‐efficacy is important for personalized interventions and improving symptom management efficiency (Ye et al. 2025). In China, an intervention based on the Omaha System framework was associated with improved sleep quality and reduced negative emotions among patients with middle‐ to late‐stage lung cancer (Bai et al. 2024).

Patients receiving ICIs may experience sleep disturbance in the context of poorer performance status and greater treatment and disease burden (Aktepe et al. 2025; Balachandran et al. 2023). The available evidence is cross‐sectional and does not establish temporality, causality or independent prediction of progression‐free or overall survival (Aktepe et al. 2025; Balachandran et al. 2023). Proposed biological explanations include inflammatory and circadian mechanisms; however, these mechanisms and their relationship to ICI outcomes require prospective investigation (Aktepe et al. 2025; Balachandran et al. 2023; Zarogoulidis et al. 2023). Given the expanding clinical use of immune checkpoint inhibitors and the high prevalence of sleep disturbances in this population, these knowledge gaps regarding integrated sleep and pain management should not be ignored.

### 3.8 Quality of Life and Patient Satisfaction

Quality‐of‐life findings from Liu and Guo's (2025) personalized nursing cohort are reported under Cognitive‐behavioural and integrated interventions. Tolstrup et al. (2020) reported that all surveyed respondents recommended the eHealth reporting tool to other patients and wished to continue using it. These responses indicate acceptability rather than proof of symptom improvement. Kwok et al. (2022) reported symptom benefits in some included studies, whereas quality‐of‐life and healthcare utilization findings were inconsistent.

### 3.9 Health Services Utilization

In the ICI‐specific trial by Zhang et al. (2022), serious immune‐related adverse events occurred in 20.6% of the ePRO group and 33.6% of the control group (HR 0.51; 95% CI 0.30–0.88; *p* = 0.01). Emergency department visits occurred in 16.3% versus 29.9% (HR 0.46; 95% CI 0.26–0.81; *p* = 0.01), and treatment discontinuation occurred in 3.6% versus 11.0% (HR 0.30; 95% CI 0.11–0.85; *p* = 0.02). Mean time per follow‐up session was 8.2 ± 3.9 min with ePRO monitoring and 36.1 ± 15.3 min with traditional follow‐up (*p* < 0.001). In a broader oncology review by Kwok et al. (2022), findings on healthcare utilization were inconsistent, and reductions in hospitalizations or unscheduled clinic visits were not consistently demonstrated.

### 3.10 Implementation Factors and Future Research

Specialized oncology nurses play an important role in ICI toxicity education, assessment, monitoring, nurse‐led follow‐up and triage; however, evidence regarding effectiveness across community and non‐specialized settings remains limited (Mirzadeh et al. 2025). Zhang et al. (2022) required participants to use a smartphone or computer, independently or with caregiver assistance. This eligibility requirement may limit applicability to patients without adequate digital access or support; the study did not quantify a 7.3% exclusion rate attributable to digital inability. Inadequate integration of PRO data into clinical workflows was another limiting factor (Tolstrup et al. 2020). Single‐center study designs may limit generalizability to lower resource settings (Liu and Guo 2025).

## 4 Discussion

This scoping review mapped the available evidence and did not undertake a formal risk‐of‐bias assessment. Consequently, the reported findings should be interpreted as a description of the direction and characteristics of the evidence rather than as a quality‐weighted conclusion regarding intervention effectiveness.

This discussion is organized around two streams of evidence: direct evidence from ICI‐treated populations and transferable evidence from broader oncology populations. Broader oncology literature is referenced only when it helps interpret the identified evidence gap or identify cautiously transferable approaches; it should not be interpreted as direct evidence of effectiveness for patients receiving ICIs. These bodies of evidence are discussed here only insofar as they inform interpretation of the ICI‐specific and transferable evidence identified in this review, rather than as direct findings about ICI‐treated patients.

The principal sleep‐related gap concerns assessment specificity rather than a complete absence of sleep‐related reporting. Explicit sleep findings in the primary studies by Bu et al. (2025) and Liu and Guo (2025), sleep‐related findings summarized by Kwok et al. (2022) and sleep items embedded in general HRQoL instruments reviewed by Hall et al. (2019) represent different forms of assessment and reporting and should not be treated as equivalent. The lack of a dedicated, validated sleep measure in the charted outcomes limits characterization of sleep disturbance and comparison across the included sources.

The broader‐oncology findings reported by Bu et al. (2025) and Liu and Guo (2025) may inform ICI nursing care processes, including structured symptom assessment, individualized education and planned follow‐up. Their applicability is limited by observational designs, mixed treatment populations and the absence of separately reported ICI‐specific intervention effects. Without an appropriate comparison, temporal symptom improvement cannot be attributed to nursing care and favourable outcomes in a general lung cancer cohort do not establish effectiveness during ICI treatment.

The broader‐oncology reviews by Park and Lee (2022), Kwok et al. (2022) and Yan et al. (2025) identify educational, physical and telehealth approaches that may inform supportive nursing care, but they do not establish effectiveness during ICI treatment. Their potential transferability depends on the targeted symptom, the feasibility of the nursing delivery model, and compatibility with the ICI‐related assessment and escalation responsibilities described by Mirzadeh et al. (2025). Variation in intervention protocols, outcome measures and follow‐up limits conclusions about the durability and reproducibility of benefits. These approaches should therefore be considered candidates for adaptation and prospective testing rather than established ICI‐specific interventions.

### 4.1 How Innovative Technologies May Transform Healthcare?

The ICI‐specific trial by Zhang et al. (2022) provides promising evidence for structured ePRO follow‐up, but its findings do not establish the effectiveness of a sleep‐targeted intervention. Its nursing relevance lies in linking symptom reporting to timely assessment, escalation and follow‐up rather than collecting PRO data alone. Tolstrup et al. (2020) further highlight the importance of clinicians reviewing and using submitted reports. Implementation should therefore specify who is responsible for reviewing reports, documenting escalation decisions and communicating clinically significant findings to the treating team. Replication in other populations and care settings remains necessary.

### 4.2 Comparison With Previous Evidence

Previous reviews have examined nursing support for cancer pain (Morikawa et al. 2023), nurse‐led telehealth during systemic or radiation therapy (Kwok et al. 2022), PROs in checkpoint inhibitor trials (Hall et al. 2019) and sleep or symptom‐cluster interventions in broader oncology populations (Sheikh‐Wu et al. 2020; Munir et al. 2025; Nissen et al. 2024). In contrast, this review maps these domains specifically for adults receiving immune checkpoint inhibitors and explicitly separates direct evidence from potentially transferable oncology evidence. Its additional contribution is not a new estimate of intervention effectiveness; rather, it demonstrates that direct immune checkpoint inhibitor–specific nursing intervention evidence remains sparse, sleep is rarely assessed using validated sleep‐specific instruments and no included intervention primarily targeted sleep disturbance in this population. The review therefore confirms the fragmentation of current symptom‐management evidence while identifying a specific measurement and intervention gap relevant to oncology nursing.

### 4.3 Implications for Nursing Practice

- Assess pain and sleep routinely: At baseline and during each ICI treatment cycle, oncology nurses should assess pain and sleep separately using standardized instruments (Balachandran et al. 2023; Mirzadeh et al. 2025). Until ICI‐specific evidence becomes available, sleep screening should be used to identify unmet needs and guide referral rather than to imply that a particular sleep intervention has proven effectiveness in this population.
- Use validated sleep‐specific measures: Where feasible, nurses should use a validated sleep‐specific measure, such as the PSQI or ISI, rather than relying only on general quality‐of‐life tools or a single insomnia item within a symptom checklist (Balachandran et al. 2023; Nissen et al. 2024).
- Develop responsive ePRO pathways: ePRO systems should include a defined process for nurse review, clinical escalation, documentation and communication with the oncology team (Zhang et al. 2022; Tolstrup et al. 2020; Mirzadeh et al. 2025).
- Apply transferable pain interventions cautiously: Educational, psycho‐educational, telehealth and non‐pharmacological pain approaches from broader oncology evidence may inform local practice, but their effectiveness should not be assumed to be identical in ICI‐treated populations (Park and Lee 2022; Kwok et al. 2022).
- Address digital inequity: Alternative non‐digital or assisted reporting options should be available for patients with limited digital literacy, limited device access, visual impairment, language barriers or other accessibility needs (Zhang et al. 2022; Mirzadeh et al. 2025).
- Prioritize ICI‐specific research: Nurse‐led trials should test integrated pain–sleep interventions in ICI‐treated populations using validated sleep measures, predefined symptom outcomes and follow‐up long enough to assess sustainability.

### 4.4 Limitations

Broadening eligibility after the exploratory search was a post hoc methodological modification. Although the transferability criteria were defined before formal full‐text screening, they were not prespecified in a registered protocol and may have introduced selection subjectivity. The inclusion of a structured clinical perspective/review also increased design heterogeneity. To reduce the risk of overinterpretation, we categorized and reported direct immune checkpoint inhibitor–specific evidence and potentially transferable oncology evidence separately throughout the review. Four of the 10 included sources were specific to ICI care: the randomized clinical trial by Zhang et al. (2022), the mixed‐methods study by Tolstrup et al. (2020), the systematic review by Hall et al. (2019) and the clinical perspective/review by Mirzadeh et al. (2025). These sources should not be interpreted as four primary evaluations of nurse‐led interventions. The remaining six sources contributed potentially transferable broader‐oncology evidence rather than direct evidence of intervention effectiveness during ICI treatment (Park and Lee 2022; Bu et al. 2025; Kwok et al. 2022; Li et al. 2023; Yan et al. 2025; Liu and Guo 2025). This distinction limits the strength of conclusions that can be drawn for ICI‐specific nursing practice. Key limitations include the heterogeneity of study designs, cancer types and outcome measures precluding meta‐analysis; geographic concentration (50% from China) limiting generalizability (Bu et al. 2025; Zhang et al. 2022; Li et al. 2023; Yan et al. 2025; Liu and Guo 2025); predominance of tertiary hospital settings with limited evidence for community care; lack of formal quality assessment; and English‐language restriction potentially missing relevant studies. In addition, short follow‐up durations (most ≤ 8 weeks, with only one study achieving 6 months) represent a critical limitation given that ICI therapy typically continues for months to years, with subsequent lines of treatment being common in melanoma and other malignancies. This temporal mismatch between intervention follow‐up and treatment duration limits our ability to assess long‐term sustainability and cumulative symptom burden management. The predominantly short follow‐up periods may underestimate intervention attenuation over time and fail to capture late‐onset sleep disturbances or chronic pain patterns characteristic of prolonged immunotherapy. Future studies should incorporate follow‐up periods extending beyond 6 months to align with real‐world ICI treatment durations and adequately evaluate intervention sustainability. In addition, no formal critical appraisal was conducted; therefore, the findings should be interpreted as descriptive evidence mapping rather than as a quality‐weighted assessment of intervention effectiveness.

## 5 Conclusion

Direct evidence regarding nurse‐led interventions for pain and sleep disturbance among patients receiving immune checkpoint inhibitors remains limited. The available immune checkpoint inhibitor–specific evidence primarily concerns electronic symptom monitoring rather than interventions designed specifically for pain or sleep. Evidence from broader oncology populations identifies potentially transferable approaches, but it should not be interpreted as proof of effectiveness during immune checkpoint inhibitor treatment. Future studies should test nurse‐led pain and sleep interventions directly in this population using validated sleep‐specific instruments, clearly defined outcomes, accessible delivery models and sufficiently long follow‐up. This review should therefore be interpreted as an evidence map for clinical awareness and research planning rather than as a recommendation of comparative intervention effectiveness.

## Author Contributions

**Omar Alqaisi:** conceptualization, methodology, validation, writing – original draft, writing – review and editing, data curation. **Mohammed Dibas:** conceptualization, project administration, writing – review and editing, methodology. **Safia Samir Darwish:** writing – review and editing, conceptualization, methodology. **Amaechi Dennis:** writing – review and editing, methodology. **Patricia Tai:** supervision, writing – review and editing.

## Funding

This scoping review received no specific funding.

## Disclosure

An earlier version of this manuscript was posted on Preprints.org on 4 January 2026 (doi: 10.20944/preprints202601.0126.v1). The manuscript has since undergone substantial revision, and the present version differs materially from the posted preprint.

## Ethics Statement

Ethical approval was not required for this scoping review, as it is based entirely on analysis of previously published literature. No human participants were directly involved; therefore, informed consent was not applicable.

## Consent

The authors have nothing to report.

## Conflicts of Interest

The authors declare no conflicts of interest.

## Supporting information


**Table S1:** (PRISMA‐ScR) checklist.
**Table S2:** Database‐specific search strategies used in the scoping review.
**Table S3:** Report retrieval status and reasons for exclusion after full‐text eligibility assessment.

## Data Availability

The authors have nothing to report.
