# Supplementary material for: Nursing Interventions for Pain and Sleep Management in Cancer Patients Receiving Immune Checkpoint Inhibitors: A Scoping Review
Source: Nurs Open. 2026 Nov 10;13(11):e71022. doi: 10.1002/nop2.71022 (PMC13750823; doi:10.1002/nop2.71022)
Supplement: Supplementary file 1 [file NOP2-13-e71022-s001.docx]

**Table S1. (PRISMA-ScR) Checklist**

| **SECTION** | **ITEM** | **PRISMA-ScR CHECKLIST ITEM** | **REPORTED ON PAGE #** |
| --- | --- | --- | --- |
| **TITLE** | | | |
| Title | 1 | Identify the report as a scoping review. | Tite page |
| **ABSTRACT** | | | |
| Structured summary | 2 | Provide a structured summary that includes (as applicable): background, objectives, eligibility criteria, sources of evidence, charting methods, results, and conclusions that relate to the review questions and objectives. | Abstract section |
| **INTRODUCTION** | | | |
| Rationale | 3 | Describe the rationale for the review in the context of what is already known. Explain why the review questions/objectives lend themselves to a scoping review approach. | Introduction |
| Objectives | 4 | Provide an explicit statement of the questions and objectives being addressed with reference to their key elements (e.g., population or participants, concepts, and context) or other relevant key elements used to conceptualize the review questions and/or objectives. | Introduction |
| **METHODS** | | | |
| Protocol and registration | 5 | Indicate whether a review protocol exists; state if and where it can be accessed (e.g., a Web address); and if available, provide registration information, including the registration number. | Methods |
| Eligibility criteria | 6 | Specify characteristics of the sources of evidence used as eligibility criteria (e.g., years considered, language, and publication status), and provide a rationale. | Methods |
| Information sources* | 7 | Describe all information sources in the search (e.g., databases with dates of coverage and contact with authors to identify additional sources), as well as the date the most recent search was executed. | Methods |
| Search | 8 | Present the full electronic search strategy for at least 1 database, including any limits used, such that it could be repeated. | Supplementary material |
| Selection of sources of evidence† | 9 | State the process for selecting sources of evidence (i.e., screening and eligibility) included in the scoping review. | Methods |
| Data charting process‡ | 10 | Describe the methods of charting data from the included sources of evidence (e.g., calibrated forms or forms that have been tested by the team before their use, and whether data charting was done independently or in duplicate) and any processes for obtaining and confirming data from investigators. | Methods |
| Data items | 11 | List and define all variables for which data were sought and any assumptions and simplifications made. | Methods |
| Critical appraisal of individual sources of evidence§ | 12 | If done, provide a rationale for conducting a critical appraisal of included sources of evidence; describe the methods used and how this information was used in any data synthesis (if appropriate). | Methods |
| Synthesis of results | 13 | Describe the methods of handling and summarizing the data that were charted. | Methods |
| **RESULTS** | | | |
| Selection of sources of evidence | 14 | Give numbers of sources of evidence screened, assessed for eligibility, and included in the review, with reasons for exclusions at each stage, ideally using a flow diagram. | Results |
| Characteristics of sources of evidence | 15 | For each source of evidence, present characteristics for which data were charted and provide the citations. | Results |
| Critical appraisal within sources of evidence | 16 | If done, present data on critical appraisal of included sources of evidence (see item 12). | NA |
| Results of individual sources of evidence | 17 | For each included source of evidence, present the relevant data that were charted that relate to the review questions and objectives. | Results |
| Synthesis of results | 18 | Summarize and/or present the charting results as they relate to the review questions and objectives. | Results |
| **DISCUSSION** | | | |
| Summary of evidence | 19 | Summarize the main results (including an overview of concepts, themes, and types of evidence available), link to the review questions and objectives, and consider the relevance to key groups. | Discussion |
| Limitations | 20 | Discuss the limitations of the scoping review process. | Limitation |
| Conclusions | 21 | Provide a general interpretation of the results with respect to the review questions and objectives, as well as potential implications and/or next steps. | Conclusion |
| **FUNDING** | | | |
| Funding | 22 | Describe sources of funding for the included sources of evidence, as well as sources of funding for the scoping review. Describe the role of the funders of the scoping review. | Declaration sections |

**Table S2. Database-Specific Search Strategies Used in the Scoping Review**

| **Database** | **Search strategy** | **Limits applied** | **Records retrieved** |
| --- | --- | --- | --- |
| **PubMed/MEDLINE** | (("Nursing"[Mesh] OR "Nursing Care"[Mesh] OR nursing[tiab] OR "oncology nursing"[tiab] OR nurse-led[tiab] OR "nursing intervention*"[tiab] OR "nursing care"[tiab] OR "nursing management"[tiab]) AND (cancer[tiab] OR neoplasm*[tiab] OR oncolog*[tiab]) AND ("immune checkpoint inhibitor*"[tiab] OR immunotherap*[tiab] OR "cancer treatment"[tiab]) AND (pain[tiab] OR "pain management"[tiab] OR sleep[tiab] OR insomnia[tiab] OR "sleep disturbance*"[tiab] OR "sleep quality"[tiab] OR "symptom management"[tiab] OR "patient-reported outcome*"[tiab])) | English language; adults; publication date: 1 January 2019 to 30 November 2025 | **146** |
| **CINAHL via EBSCOhost** | (MH "Oncologic Nursing" OR MH "Nursing Care" OR TI nursing OR AB nursing OR TI "oncology nursing" OR AB "oncology nursing" OR TI nurse-led OR AB nurse-led OR TI "nursing intervention*" OR AB "nursing intervention*" OR TI "nursing care" OR AB "nursing care" OR TI "nursing management" OR AB "nursing management") AND (TI cancer OR AB cancer OR TI neoplasm* OR AB neoplasm* OR TI oncolog* OR AB oncolog*) AND (TI "immune checkpoint inhibitor*" OR AB "immune checkpoint inhibitor*" OR TI immunotherap* OR AB immunotherap* OR TI "cancer treatment" OR AB "cancer treatment") AND (TI pain OR AB pain OR TI "pain management" OR AB "pain management" OR TI sleep OR AB sleep OR TI insomnia OR AB insomnia OR TI "sleep disturbance*" OR AB "sleep disturbance*" OR TI "sleep quality" OR AB "sleep quality" OR TI "symptom management" OR AB "symptom management" OR TI "patient-reported outcome*" OR AB "patient-reported outcome*") | English language; adults; publication date: 1 January 2019 to 30 November 2025 | **92** |
| **Scopus** | TITLE-ABS-KEY (nursing OR "oncology nursing" OR "nurse-led" OR "nursing intervention*" OR "nursing care" OR "nursing management") AND TITLE-ABS-KEY (cancer OR neoplasm* OR oncolog*) AND TITLE-ABS-KEY ("immune checkpoint inhibitor*" OR immunotherap* OR "cancer treatment") AND TITLE-ABS-KEY (pain OR "pain management" OR sleep OR insomnia OR "sleep disturbance*" OR "sleep quality" OR "symptom management" OR "patient-reported outcome*") | English language; publication years 2019–2025; searched through 30 November 2025 | **157** |
| **ScienceDirect** | (nursing OR "oncology nursing" OR "nurse-led" OR "nursing intervention" OR "nursing care" OR "nursing management") AND (cancer OR neoplasm OR oncology) AND ("immune checkpoint inhibitor" OR immunotherapy OR "cancer treatment") AND (pain OR "pain management" OR sleep OR insomnia OR "sleep disturbance" OR "sleep quality" OR "symptom management" OR "patient-reported outcome") | English language; publication date: 1 January 2019 to 30 November 2025 | **90** |
| **Total** |  |  | **485** |

**Search date:** 30 November 2025.
**Date range:** 1 January 2019 to 30 November 2025.
**Language restriction:** English.
**Records identified before duplicate removal:** 485.

**Table S3. Report retrieval status and reasons for exclusion after full-text eligibility assessment**

**A. Reports not retrieved**

| **Retrieval status** | **Number of reports** |
| --- | --- |
| Full text unavailable | 2 |
| **Total reports not retrieved** | **2** |

**B. Reports excluded after full-text eligibility assessment**

| **Reason for exclusion** | **Number of reports** |
| --- | --- |
| No nurse-led intervention or nursing-care framework | 14 |
| Did not report pain, sleep, or a relevant symptom-management outcome | 9 |
| Population not receiving ICIs and not transferable to ICI care | 8 |
| Protocol, conference abstract, editorial, or narrative article without an explicit nursing framework | 6 |
| Focused exclusively on pharmacological management | 2 |
| **Total reports excluded after eligibility assessment** | **39** |
